# Symmetry Breaking in Meniscus Splitting: Effects of Boundary Conditions and Polymeric Membrane Growth

**DOI:** 10.1002/advs.202503807

**Published:** 2025-06-03

**Authors:** Thi Kim Loc Nguyen, Taisuke Hatta, Koji Ogura, Yoshiya Tonomura, Kosuke Okeyoshi

**Affiliations:** ^1^ Graduate School of Advanced Science and Technology Japan Advanced Institute of Science and Technology 1‐1 Asahidai Nomi Ishikawa 923–1292 Japan

**Keywords:** capillary length, chitosan, interface, polysaccharides, symmetry breaking

## Abstract

An evaporative interface creates a non‐equilibrium state for both dissolved molecules and geometrically asymmetric macrostructures such as those involved in crystal growth. The phenomenon of meniscus splitting has been discovered and hydrodynamically demonstrated using a Hele–Shaw cell by drying a viscous polymer solution/dispersion. The generation of multiple nuclei has been reported; however, the relative nuclei positions and dominant factors remain unclear. This study investigates meniscus splitting involving multiple nucleation events, focusing on symmetry breaking and asynchronous nucleus formation. Fundamentally, it is found that rather than splitting into halves or thirds, the splitting is uneven. With the cell width setting the boundary conditions, the nucleus is not generated at the center of the cell width, but rather is shifted to an asymmetric position, as shown by the statistical analysis of the nuclei positions. The shift strongly facilitates asynchronous second nucleation. Such deviations from symmetry and synchrony are crucial for interpreting dissipative structures and their time evolution.

## Graphical abstract

Meniscus splitting in the drying of a viscous polymer dispersion is investigated by focusing on nucleation of multiple nuclei with symmetry breaking and asynchronous nucleus formation. Controlled by spatial boundaries, the first nucleus is generated at a position shifted away from the space center and strongly facilitates the asynchronous second nucleation. Such deviations from symmetry and synchrony are crucial for interpreting dissipative structures and their time evolution.

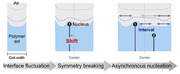

## 1 Introduction

**Figure 1:**
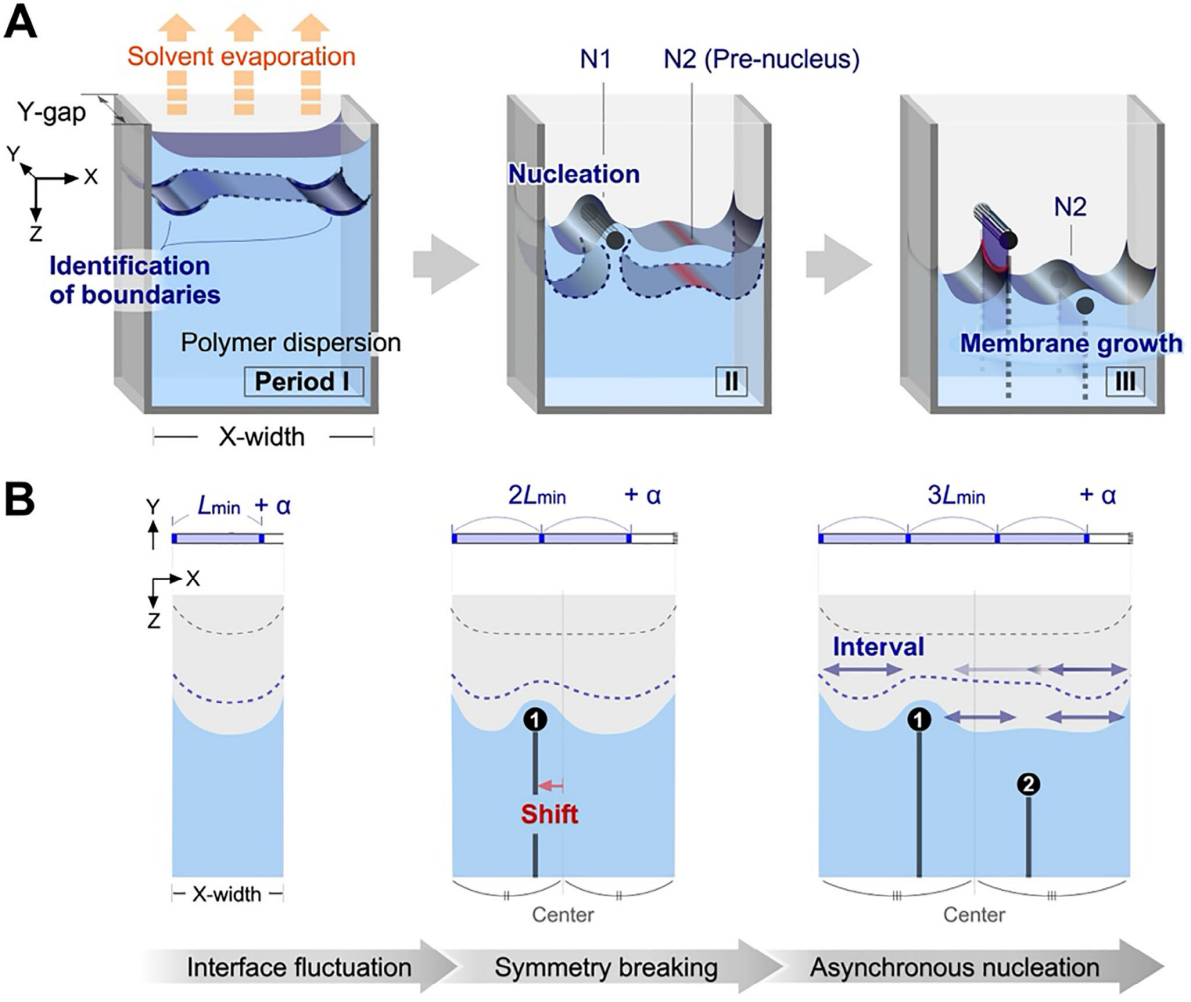
Schematic of meniscus splitting through symmetry breaking. A) Aqueous polymer dispersion in a Hele–Shaw cell is affected by sidewalls acting as boundaries during evaporation. This occurs due to the interface fluctuations of the polymer, which increase the area for water evaporation. An inhomogeneous concentration at the interface leads to the generation of the first nucleus (N1). In cells with sufficient width, a second nucleus (N2) forms to bridge the gap. B) Hypothesis suggesting that the spatial constraint imposed by cell width influences nuclei positions and stepwise nucleation. As the cell width increases, interface fluctuations, symmetry breaking, and asynchronous nucleation become increasingly prominent. *L*_min_ indicates the minimum length for the intervals observed in experiments, while *α* denotes lengths shorter than *L*_min_.

In non‐equilibrium drying environments in nature, geometric patterns occur through self‐organization, such as the spirals seen in flowers. While the growth of these patterns has been explained, an understanding of the effects of symmetry/asymmetry on macroscopic geometric structures is still lacking.^[^ ^1^, ^2^, ^3^, ^4^, ^5^, ^6^, ^7^, ^8^, ^9^ ^]^ From a soft matter perspective, the generation of patterns under physicochemically controlled environments, such as the Saffman–Taylor instability that induces viscous fingering patterns, is feasible.^[^ ^10^, ^11^, ^12^, ^13^, ^14^, ^15^, ^16^, ^17^, ^18^, ^19^, ^20^ ^]^ Usually, the typical fingering patterns are discussed using a fluid sandwiched between two substrates without boundaries.^[^ ^14^ ^]^ Through the exploration of transient phenomena, we have identified and studied a phenomenon known as “meniscus splitting.”^[^ ^13^, ^14^, ^15^, ^16^, ^17^, ^18^, ^19^, ^20^, ^21^, ^22^, ^23^, ^24^, ^25^, ^26^ ^]^ Unlike previously reported dissipative structures that transiently exhibit geometric patterns, we successfully identified the environments that support dissipative structures with the formation of polymeric membranes. By evaporating a polymer solution/dispersion in a top‐open space with a narrow gap, the polymer is deposited to bridge the gap at specific positions and divide the evaporating meniscus into multiple segments (**Figure** **1**). The local polymer concentration at the evaporative interface increases significantly, leading to interface fluctuations during solvent evaporation. The critical condition is that the gap must be less than a capillary length,^[^ ^27^, ^28^ ^]^ which is typically ∼2 mm, and the splitting phenomenon occurs on various hydrophilic/hydrophobic substrates such as glass, plastics, and metals.^[^ ^29^, ^30^, ^31^, ^32^, ^33^, ^34^ ^]^ By adjusting the initial polymer concentration, drying temperature, and relative humidity, we observed splitting with a characteristic interval length of ∼10 mm between the nuclei. This length was largely consistent across different types of polysaccharides, resulting in the formation of self‐assembled microfibers or microparticles.^[^ ^35^, ^36^, ^37^, ^38^, ^39^, ^40^ ^]^ Furthermore, the deposited polysaccharide membranes are useful as anisotropic soft materials that respond to the water vapor in the air or to the variations in the water pH and in the type of cations in the water.^[^ ^40^, ^41^, ^42^, ^43^, ^44^, ^45^, ^46^, ^47^ ^]^

In this study, we report that meniscus splitting has physical significance, particularly under conditions for the formation of multiple nuclei. Previous studies for the typical fingering patterns have discussed the averaged characteristic wavelength or treated the system using symmetric geometries. These treatments are based on the setting of arbitrary positions in infinite space. By contrast, we focus on the asymmetry generated by a space with clear finite dimensions. In particular, the meniscus splitting into two segments is compared to the splitting into three segments. Figure 1A illustrates the splitting of the meniscus into three segments through the generation of two nuclei. While multiple nucleations have been reported in previous work, the underlying mechanisms of this process remain unclear. To validate this process, we focused on the stepwise changes in the interfacial fluctuation, highlighting the identification of boundaries (period I) and subsequent stages. Two primary periods were identified: the first nucleation (N1) with symmetry breaking (period II) and the second nucleation (N2) with asynchronous nucleation (period III). Spatial off‐center shifts of the nuclei are commonly observed in experiments, and it is important to consider this phenomenon. As the cell width increased, the position of the nucleus shifted along the width direction (*X*‐direction), and the meniscus split into two or three unequal segments rather than splitting into equal segments (Figure 1B). The shift of the *X*‐position of N1 from the center along the *X*‐direction signifies symmetry breaking. Additionally, the splitting of the meniscus into three segments has distinct implications compared to the splitting into two segments. The difference in the depth (position along *Z*‐direction) between N1 and N2 suggests asynchronous deposition. During the three periods, a critical distance for the interval between the nuclei appears to emerge. Based on these results, nucleation is accompanied by symmetry breaking and asynchronous nucleation. To elucidate the positions of the nuclei in physical spaces, the effect of the cell width on the aqueous chitosan dispersions was examined. Notably, the nucleation phenomena were statistically analyzed by assessing the probability of the number of split interfaces, positions of one‐nucleus/two‐nuclei, and distributions of the nuclei.

## 2 Results and Discussion

**Figure 2:**
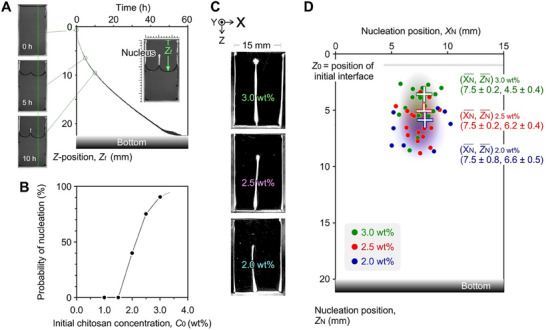
Effect of the initial chitosan concentration on deposition nucleation. A) Time evolution of the air‐liquid interface and nucleation process. B) Nucleation probability as a function of initial polymer concentration, based on at least 20 trials for each condition. C) Photograph of the deposited vertical membrane formed by drying polymer dispersion with different initial chitosan concentrations from a top‐side‐open cell (15, 0.5, ∼23 mm). Initial acetic acid concentration: 1.0 vol%. Drying temperature: 40 °C. D) Statistical analysis of the nucleus position (*X*_N_, *Z*_N_). Circles represent *XZ*‐positions observed in individual trials, while crosses denote the average *XZ*‐positions.

For the drying experiment, aqueous dispersions of chitosan with a molecular weight (*M*_w_) of 50–190 kDa were poured into a top‐side open cell designed as a Hele–Shaw cell with a width of 15 mm. The sample was positioned under atmospheric pressure in a 1‐L box with an air circulator at a constant temperature of ∼40 °C and a constant relative humidity of 20–30% (see Figure S1, Supporting Information). The drying process was observed using cross‐polarized light with a first retardation plate (*λ* = 530 nm) to monitor the time evolution of the interface position. **Figure** **2**A shows the time evolution of the *Z*‐position of the evaporative meniscus on the green line in the inset image (*Z*_t_). Nucleation initiates near the center of the *X*‐width, leading to the splitting of the meniscus into two segments. For *Z*_t_, the time evolution appeared to be smooth immediately before and after nucleation (∼5 h). After the nucleus position is fixed, the rate of change in *Z*_t_ gradually decreases for 10–15 h. This deceleration occurs because the evaporative interface expands around the nucleus while the bottom of the split interface does not move. Thermal energy is used for solvent evaporation by depositing the condensed polymer at a specific position.

Next, to elucidate the effect of the initial chitosan concentration on meniscus splitting, the spatial position of the evaporative interface and the deposition nucleus were statistically analyzed by performing more than 20 trials for each condition. The probability of nucleation increased significantly when the chitosan concentration *C*_0_ reached 2.0 wt.% (Figure 2B), and the formation of vertical membranes was clearly enhanced for *C*_0_ ≥ 2.0 wt.% (Figure 2C). This drastic change indicates that *C*_0_ is a key factor for the splitting phenomena, which also requires appropriate physical parameters, such as the evaporation rate.^[^ ^47^, ^48^, ^49^ ^]^ For example, the chitosan concentration increases from 1 to 3 wt.% when the volume is reduced to one‐third, but the probability of nucleation is ∼0%. Statistical analysis suggests that nucleation requires the combination of a chitosan concentration close to the saturating concentration and a high evaporation rate.

Figure 2D shows the distribution of the nucleation positions (*X*_N_, *Z*_N_) following the drying of the chitosan dispersion at varying initial concentrations in a cell with a width of 15 mm. The position data were collected and plotted without any normalization. Nucleation was not observed for the samples with the initial concentrations of 1.0‐ and 1.5‐wt.%. It is considered that even if pre‐nuclei are formed in the 1.0‐ and 1.5‐wt.% polymer dispersions, they do not bridge the gap to form the nuclei because the evaporation rate is not high enough in the middle of the cell depth. By contrast, the nucleation positions for the samples with the initial concentrations of 2.0‐, 2.5‐, and 3.0‐wt.% are estimated as (7.5 ± 0.8, 6.6 ± 0.5), (7.5 ± 0.2, 6.2 ± 0.4), and (7.5 ± 0.2, 4.5 ± 0.4), respectively. While *X*_N_ was mostly located close to the center for all *C*_0_, *Z*_N_ decreased with increasing *C*_0_. This finding supports the hypothesis that the polymer bridges the gap and splits the meniscus into two when the dispersion approaches the saturated concentration. Additionally, the *X*_N_ were found at positions between 5 and 10 mm without any *X*_N_ at either 0–5 or 10–15 mm ranges, suggesting that the cell walls exert a repulsive force. The balance of the interfacial tension and the capillary force is discussed below. The presence of a single nucleus suggests that the evaporative interface experiences fluctuations involving multiple clusters, akin to the pre‐nucleus, which accumulate around the center of the cell width.

**Figure 3:**
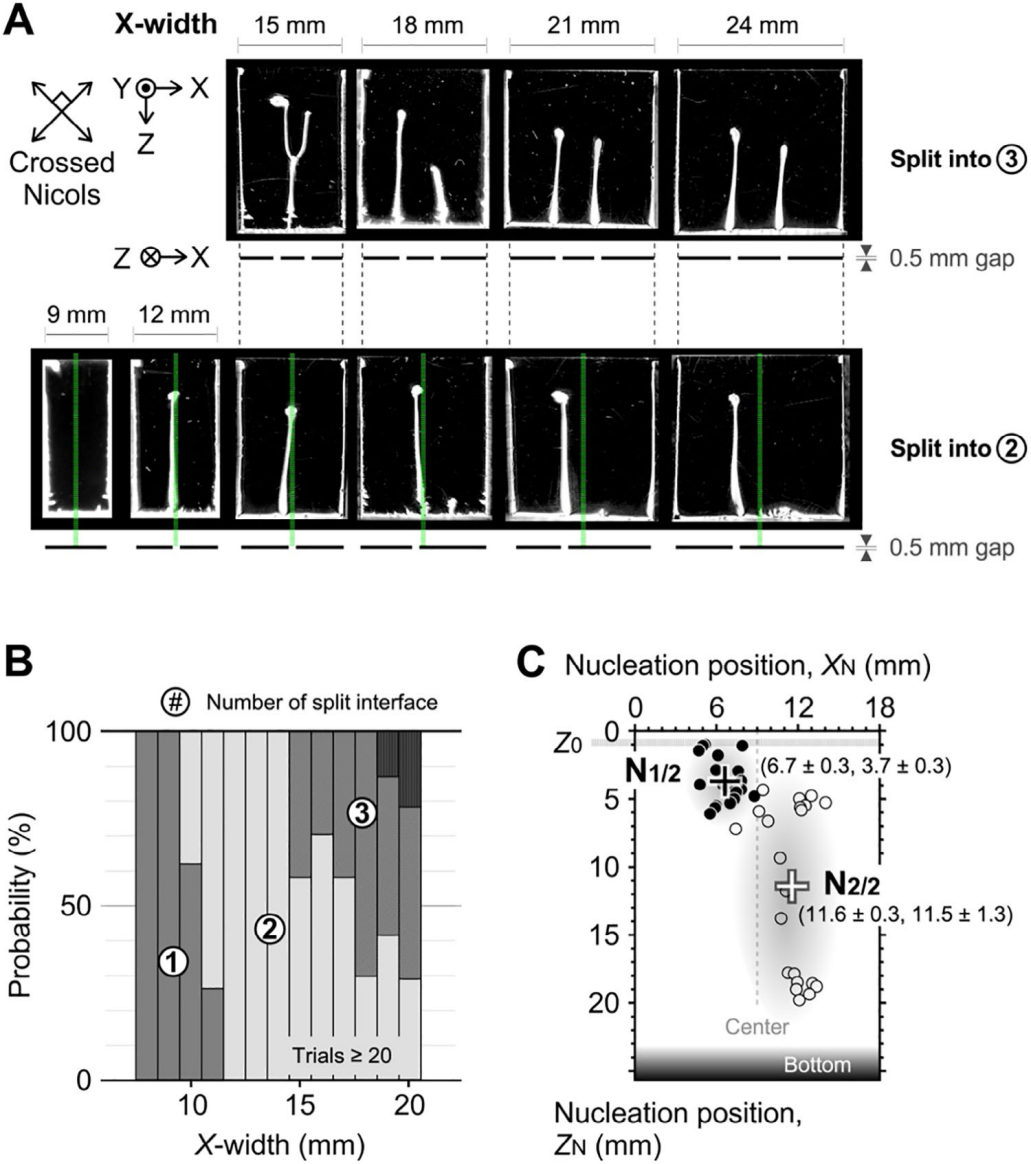
Effect of cell width on deposition nucleation. A) Images of polymer deposition in cells with specified widths. The green dotted line represents the center of the cell width, while the black horizontal line below the image corresponds to the length of the split interface. Initial chitosan concentration: 2.5 wt.%. Initial acetic acid concentration: 1.0 vol%. Cell dimensions: *X*‐width × 0.5 mm × ∼23 mm. Drying temperature: 40 °C. Numbers within circles denote the number of split interfaces observed. B) Probability of split interface formation as a function of cell width, based on more than 20 trials. C) Statistical analysis of nucleation positions (*X*_N_, *Z*_N_) for cases of single and dual nuclei formation in a cell with the dimensions of 18 mm × 0.5 mm × ∼23 mm. N_1/2_ represents the first nucleus, while N_2/2_ indicates the second nucleus.

To elucidate the effect of cell *X*‐width on nucleation, we conducted a statistical analysis of the spatial positions of the nuclei. The results of the analysis are presented in **Figure** **3**A and show that the number of nuclei and the number of split interfaces increase with increasing *X*‐width. Specifically, when a nucleus originated from the cells with an *X*‐width exceeding 15 mm, its position was not fixed at the center but rather was shifted away from the center, indicating a symmetry‐breaking process. Moreover, under similar conditions, the occurrence of two nuclei was observed with a significant probability, as demonstrated through a statistical analysis involving over 20 trials for each *X*‐width (Figure 3B). This clearly indicates that a single condition allows multiple possible outcomes for the number of menisci formed during the splitting. For instance, cells with a width greater than 15 mm can yield two menisci with a probability of ∼60% and three menisci with a probability of ∼40%. The possibility of different outcomes implies that the evaporative interface contains fluctuating polymers which play a crucial role in deposition nucleation. This scenario is further supported by the symmetry‐breaking phenomenon following boundary identification, as shown in Figure 1A. The phenomenon of multiple nucleations likely arises due to the accelerating inhomogeneity facilitating pre‐nuclei generation with increasing interface length, similar to the observations reported in our previous studies.^[^ ^47^, ^48^, ^49^, ^50^ ^]^

The nucleation positions (*X*_N_ and *Z*_N_) in a cell with a width of 18 mm were analyzed by classifying the cases into the categories of single and double nuclei (Figure 3C; Figure S2, Supporting Information). For the two nuclei with three split interfaces, the positions of the nuclei (*X*_N1/2_, *Z*_N1/2_) and (*X*_N2/2_, *Z*_N2/2_) were estimated as statistical averages. Here, the position of the first nucleus, N_1/2_, is defined as being located on the left, with the closer corner at the top serving as the origin. If N_1/2_ appeared to the right of the image center, the left and right sides were interchanged, and the analysis was conducted using the upper‐left corner as the origin. The positions for N1/2 and N2/2 were (6.7 ± 0.3, 3.7 ± 0.3) and (11.6 ± 0.3, 11.5 ± 1.3), respectively. The two *X*_N_ positions are located close to the third segment of the cell width. This implies that the cell width of 18 mm leads to splitting into three interfaces rather than two interfaces with a probability exceeding 50%. The initial interface does not split from the center (*X* = 9) but rather from the *X*‐positions near the trisection of the cell *X*‐width (*X* = 6 or 12). This indicates that the primary fluctuation at the interface plays a crucial role in determining the nucleation positions. Moreover, sequential growth is a distinctive feature of the development of two nuclei and three menisci. The *Z*‐position of the second nucleus, *Z*_N2/2_, is notably different from that of the first nucleus (*Z*_N2/2_–*Z*_N1/2_ = 7.8 mm). This discrepancy arises because the first nucleus enhances the attraction of the polymer clusters toward itself, while the second pre‐nuclei concurrently reduce this attraction. The gradual increase in the viscosity during drying delays the deposition of the second nucleus. As a result of the deceleration of the growth from the pre‐nucleus to the nucleus, *Z*_N2/2_ was widely distributed. Consequently, symmetry breaking along the *X*‐axis leads to asynchronous deposition along the *Z*‐axis during solvent evaporation.

**Figure 4:**
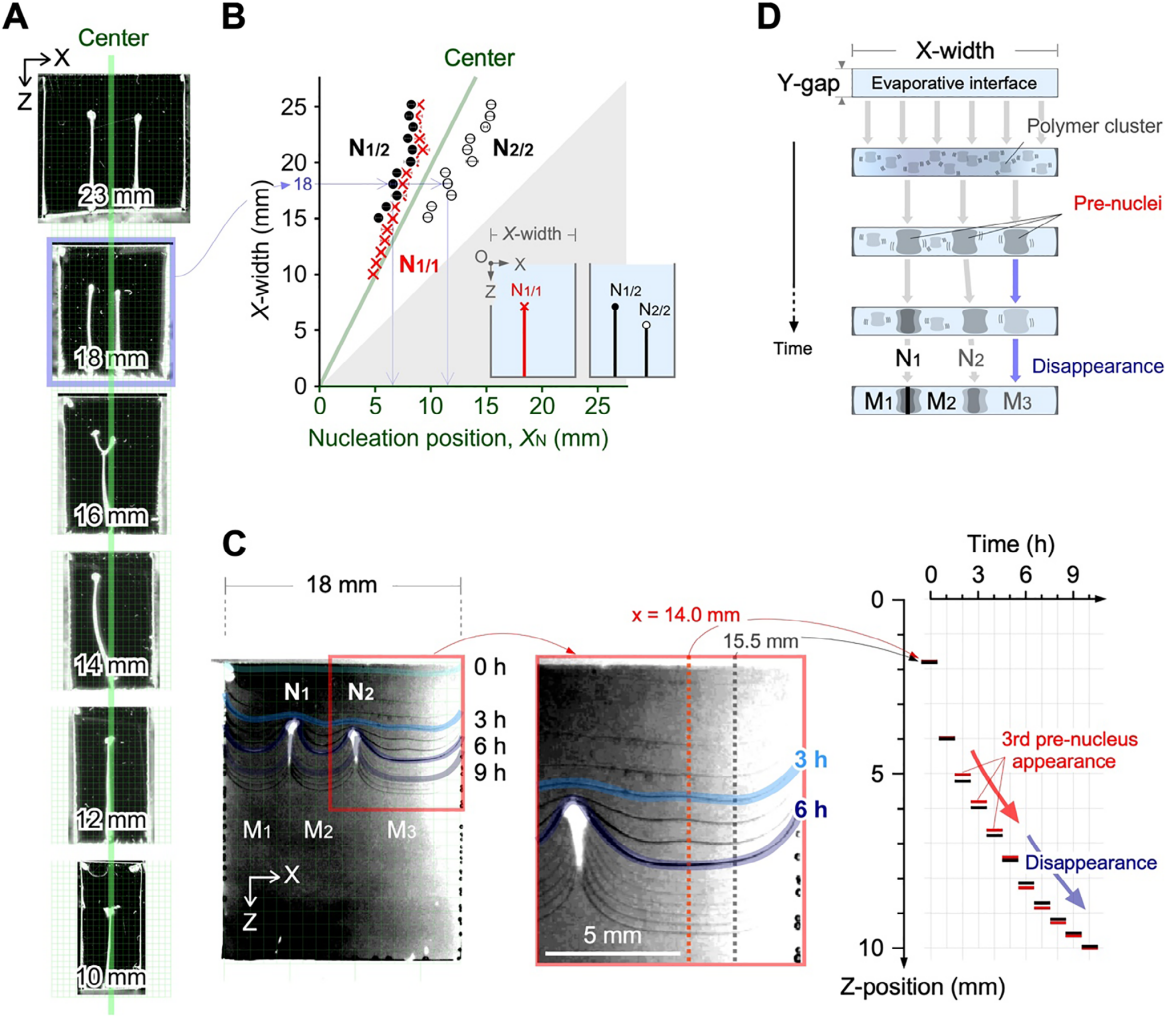
Symmetry breaking in meniscus splitting. A) Deposited membranes in cells with specified *X*‐widths. The green line indicates the center of the cell width. Initial chitosan concentration: 2.5 wt.%. Initial acetic acid concentration: 1.0 vol%. Cell dimensions: *X*‐width × 0.5 mm × ∼23 mm. Drying temperature: 40 °C. B) Statistical average of the nucleation position, *X*_N_, as a function of *X*‐width. N_1/1_ represents a nucleus for single nucleation. N_1/2_ and N_2/2_ correspond to the first and second nuclei of systems with two nucleations, respectively. More than 15 trials were conducted for each condition. C) Time‐evolution of meniscus splitting in a cell with a width of 18 mm focusing on area M3, where the appearance and disappearance of the third pre‐nucleus (M3) can be observed. D) Schematic of the temporal changes at the evaporative interface. During the induction period, polymer clusters fluctuate and form multiple pre‐nuclei that do not bridge the gap. Once a nucleus is formed, the growth of subsequent pre‐nuclei slows down, leading to asynchronous nucleation.

A comprehensive analysis of the nucleation of the meniscus splitting into two and three segments was carried out. With an increase in the *X*‐width from 10 to 20 mm, the shape of the deposited polymer transitioned from no distinct shape to the I‐, Y‐, and II‐shapes (**Figure** **4**A). In particular, the 14‐mm‐wide cell provided an I‐shaped deposition with a shift of the nucleus position from the center. This symmetry breaking was studied by plotting the statistical average *X*_N1/1_ for one nucleus and *X*_N1/2_ and *X*_N2/2_ for two nuclei versus the *X*‐width of the cell (Figure 4B; see Figure S3, Supporting Information). When the *X*‐width was less than 9 mm, no nucleation was observed; hence, there are no data points for *X*‐width less than 9 mm in the graph. By contrast, when the *X*‐width is greater than 10 mm, one nucleus position was observed that was shifted away from the center, with the degree of the shift increasing with increasing *X*‐width. Two nuclei are generated for *X*‐widths greater than 15 mm. It is noteworthy that the *X*‐positions of N_1/2_ are close to those of N_1/1_. The shift for N_1/1_ indicates symmetry breaking and induces the generation of multiple nuclei. This interpretation is supported by the fact that the *X*‐positions of N_1/2_ are closer to the center than the *X*‐positions of N_1/1_.

Focusing on the induction time period that precedes the nucleation in an 18‐mm‐wide cell, the deformation of the interfacial line indicated the presence of a pre‐nucleus (Figure 4C; see Movie S1, Supporting Information). Monitoring of the time‐evolution on the red (*x* = 14.0 mm) and black lines (*x* = 15.5 mm) showed that a small pre‐nucleus was transiently generated around the position on the red line. The difference between the red/black positions reached ∼100 µm, despite being less than 10 µm before and after this time period (2–5 h). This result clearly indicates that the polymer clusters beneath the interface form a pre‐nucleus at the *X*‐position. However, this pre‐nucleus could not become a nucleus owing to insufficient conditions. The mechanism of the evaporative interface (*XY*‐plane) is summarized in Figure 4D. The polymer clusters have almost equal possibilities of becoming pre‐nuclei during the fluctuation period. However, the boundaries of the two sidewalls initially affect the curved interfaces, affecting the initial position of the pre‐nuclei and the number of nuclei (N1 and N2). The pre‐nucleus indicates the presence of a large polymer cluster but does not bridge the gap. If the pre‐nucleus for N3 appears during the induction period, it will disappear because the polymer clusters are attracted to and are integrated into the solidified N1 or N2 with a stronger capillary force.

**Figure 5:**
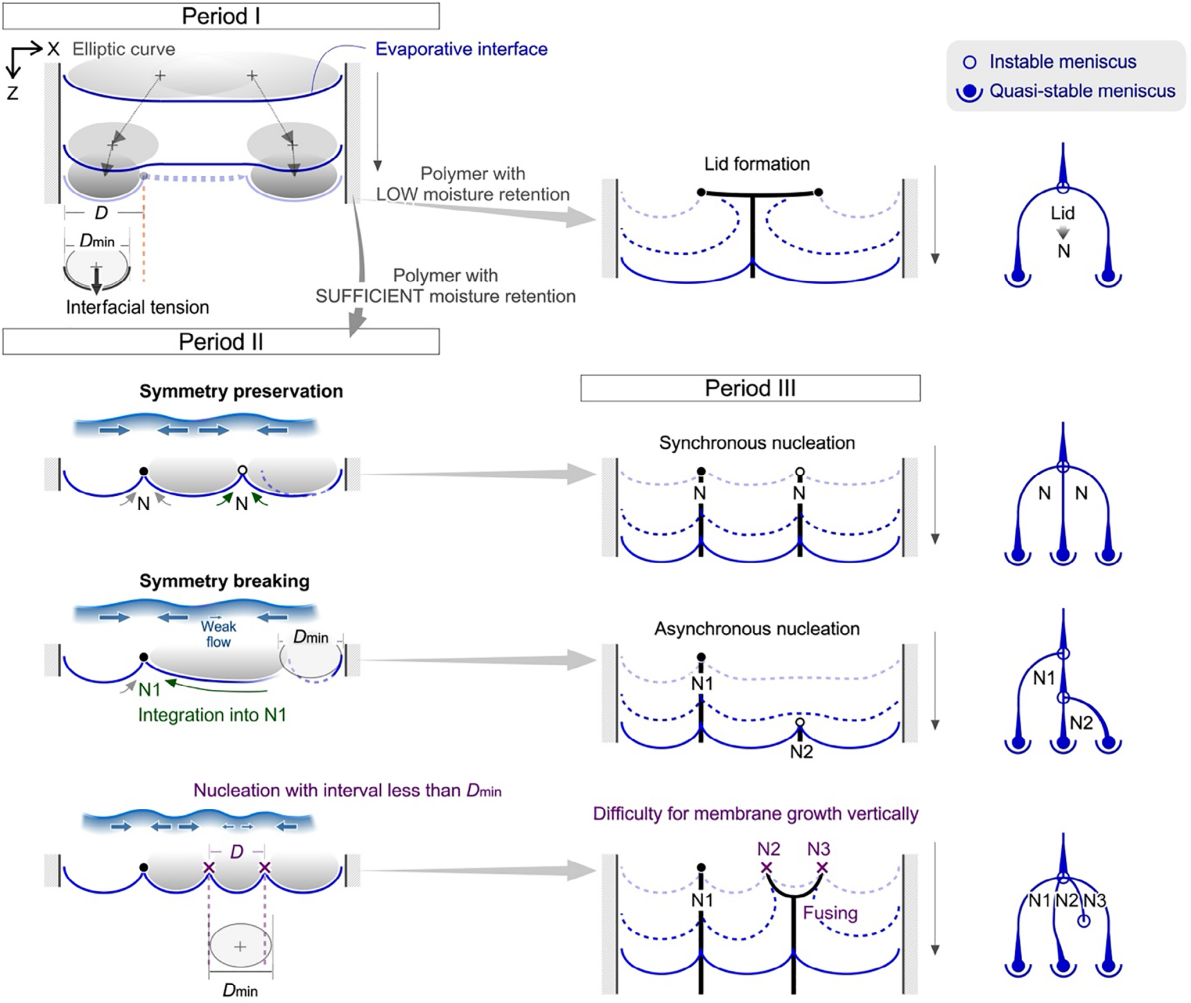
Meniscus splitting through symmetry preservation/breaking for synchronous/asynchronous nucleation. Schematics of the splitting process into three menisci with a high probability. Period I: Initial meniscus deformation near the two boundaries, forming an elliptic curve. Period II: Instable meniscus showing both symmetry preservation and symmetry breaking. Period III: Membrane growth as a quasi‐stable process. *D* represents the diameter of the ellipse fitting the interface, while *D*_min_ is the minimum diameter at which the interface can form. N1 and N2 represent the first and second nuclei, respectively. The schematics on the right show the branching model for each case.

During the splitting process, a characteristic interval along the *X*‐direction was observed between the nuclei. Interestingly, the nucleation does not start from the middle of the cell width but rather exhibits a stepwise growth. To discuss the generation of multiple nuclei, the geometric consideration of the conditions that enable the generation of the three menisci is discussed by focusing on the characteristic periods (**Figure** **5**): I) The effect of the boundaries by the deformation of the meniscus near the two side walls, II) simultaneous nucleation while preserving symmetry, or relaxation of one pre‐nucleus growth with asymmetrical nucleation, and III) membrane growth from nuclei vertically to the initial meniscus as a quasi‐stable step. The multiple pre‐nuclei grow with intervals above a certain distance and either synchronous or asynchronous nucleation occurs. On the other hand, when the multiple growing pre‐nuclei are separated by an interval below a certain distance, the boundaries of the closest two nuclei gradually come closer and the pre‐nuclei finally fuse into a single nucleus because of the strong capillary force between them. A branching model would be helpful for understanding the time evolution of the different cases. While synchronous branching preserves symmetry, asynchronous branching induces symmetry breaking.

Based on the experimental results, the concave curvature was deformed by the effect of the two boundaries in the period I. The concave curves can be approximated by elliptic curves with diameter *D* (see Figure S4, Supporting Information), which has the minimum value of *D*_min_. This value should be defined based on the capillary length, *l*_cap_, which is controlled by the interfacial tension and the density on the evaporative interface according to^[^ ^27^ ^]^

(1) $$D_{\min}\approx 2l_{\mathrm{cap}}=2\sqrt{\frac{\gamma }{g\Delta \rho }}$$

where *γ* is the interfacial tension, *g* is the gravitation acceleration, and *∆ρ* is the mass density difference between the two phases. The *D*_min_ value is approximately twice that of *l*_cap_ for typical polymer solutions/dispersions (*D*_min_ ≈ 2*l*_cap_), as follows. *l*_cap_ provides a rough estimate of the effective distance from the wall and the meniscus curve is approximated as an exponential. When the liquid is confined between two walls, it is affected by both walls. For lower distances between the two walls, the meniscus curve can be approximated as an elliptic curve. This means that the curve transitions from hyperbolic into elliptic when the distance is close to 2*l*_cap_. Therefore, 2*l*_cap_ is a natural approximation for the critical diameter of a droplet or particle. In the application of this argument in the present study, the pre‐nucleus on the interface can act as the second wall. The *l*_cap_ of water at room temperature was calculated using Equation (1) (*γ ≈* 70 mN m^−1^, *g* = 9.8 m s^−2^, Δ*ρ ≈* 1000 kg m^−3^), resulting in a value of 2.7 mm. Based on this, the typical value for the air‐water interface is in the range of 2–3 mm, which is a physical limit set by capillarity.^[^ ^27^ ^]^ Similarly, *D*_min_ can be estimated to be twice that of *l*_cap_ and thus equal to 4–6 mm. The value of *D*_min_ for the meniscus splitting in the 2.5 wt.% chitosan sample in 1 v/v% acetic acid at 40 °C was calculated from the experimental values using Equation (1) (*γ* = 67.6 mN m^−1^, *g* = 9.8 m s^−2^, *ρ*_sol_ = 1.00466 g mL^−1^, *ρ*_air_ = 1.13 kg m^−3^), resulting in a value of 5.24 mm. This value is sufficiently close to the actual intervals between the nuclei, *L*_min_, in the meniscus splitting and membrane growth.

When the polymer dispersion is dried from a cell with a narrow gap < *l*_cap_, the polymer forms clusters for deposition and bridges the gap at multiple *X*‐positions. Rapid drying and the use of polymers with low moisture retention at the interface lead to the formation of a lid in the center of the cell, causing the evaporative interface to expand at the two boundaries. Subsequently, the polymer is deposited as a membrane and grows in the *Z*‐direction without undergoing the processes of period II. To prevent lid formation, various methods can be employed, such as reducing the initial polymer concentration, decreasing the evaporation rate, and increasing the gap size.

By contrast, when the polymer retained sufficient moisture, a clear period II was distinctly observed. The evaporative interface preserved the inhomogeneity of the polymer density while it was affected by the capillary force arising from the boundary wall. This inhomogeneity enhances the generation of a wavy interface, facilitating mass/heat flows beneath the interface. Near the interfacial curves at ∼2*l*_cap_, the polymer remained stable in the *X*‐position and filled the gap. Two scenarios arise: preserving symmetry through synchronous nucleation and breaking symmetry leading to asynchronous nucleation. To maintain symmetry, the polymer clusters at two *X*‐positions simultaneously form independent nuclei with an interval exceeding *D*_min_. However, real‐world processes often lead to symmetry breaking. The polymer density beneath the interface became more inhomogeneous during the initial polymer deposition on N1. Upon N1 generation, the dense polymer around N2 is attracted to N1, causing the rate of the increase in the polymer density around the N2 to decrease. This amplification of inhomogeneity triggers symmetry breaking.

In the drying of the polymer dispersion from a cell with a width of 3*D*_min_+*α*, splitting into four interfaces was seldom observed. This is because the pre‐nuclei within *D*_min_ are repelled by interfacial tension. As a result, we observed pre‐nuclei disappearance or fusion with the adjacent nucleus (see Movie S2, Supporting Information). As an example, as shown in Figure 4C, the splitting into three rather than four nuclei can be explained by the effect of *D*_min_ (18 mm/3 = 6 mm > *D*_min_, 18 mm/4 = 4.5 mm < *D*_min_). One of the pre‐nuclei could not become a nucleus and relaxed and disappeared because the interval adjacent to the nucleus was less than *D*_min_. It is difficult for nucleation to occur with an interval of less than *D*_min_. Thus, the condition of 3*D*_min_ < *X*‐width < 4*D*_min_, such as 18 mm, allows one meniscus to settle into three split menisci.

## 3 Conclusion

In conclusion, meniscus splitting with multiple nucleations was demonstrated by focusing on deviations from symmetry and synchronicity. Using an aqueous chitosan dispersion, these phenomena were statistically analyzed and explored in detail by controlling the initial polymer concentration and the width of the Hele–Shaw cell. The study revealed that single nucleation with a shift away from the center of the cell width clearly indicated the symmetry breaking of the splitting, and the position of the deposited nucleus was asymmetric. This asymmetry is also reflected in asynchronous nucleation during the generation of two nuclei. These features arise from the non‐equilibrium condition at the evaporative interface which undergoes drying into the upper layer and wetting from underneath. The condensed polymer at the interface could undergo hydration and dehydration. Initially, after evaporation begins, multiple pre‐nuclei can grow, and some are selected or integrated into several points with an interval greater than twice the capillary length. Considering that the interface curve can be fitted by an elliptical arc, the length of the major axis of the elliptic arc is the interval between the nuclei. The interfacial tension between the air and dispersion is a significant factor affecting this interval. This simple exploration of the splitting process contributes to the fundamental understanding of symmetry breaking and the asynchronous generation of dissipative structures. In addition, by precisely controlling the drying conditions of the dispersions, the characteristic polymer deposition through solvent evaporation can be elucidated, similar to the crystal growth of small molecules. These insights pave the way for the practical applications of symmetry breaking in designing and optimizing polymer‐based materials and processes.^[^ ^42^, ^43^, ^49^ ^]^ Furthermore, the physical demonstrations carried out in this study are beneficial for the development of understanding of pattern formation by using neural networks,^[^ ^51^, ^52^ ^]^ data sciences,^[^ ^53^ ^]^ numerical simulations,^[^ ^54^ ^]^ based on mathematical sciences.

## 4 Experimental Section

### Materials

Chitosan, poly(D‐glucosamine) with a molecular weight (*M*_w_) of 50–190 kDa, was acquired from Merck KGaA (Darmstadt, Germany). The chitosan was 75–85% deacetylated, and the viscosity of the aqueous dispersion was 200–800 cP (1 wt.% in 1% acetic acid). This dispersion was known as deacetylated chitin which was categorized as a low‐molecular‐weight polymer. Acetic acid (99.7%, FUJIFILM Wako Pure Chemical Corporation, Osaka, Japan) was utilized as received. Following the dissolution of the chitosan powder in an aqueous dispersion containing acetic acid, a tiny amount of impurities and air bubbles were eliminated through centrifugation for 30 min at 25 °C and 21800 × g using a CF15RN centrifuge (Eppendorf Himac Technologies Co., Ltd, Japan) equipped with an angle rotor (Angle Rotor T15A36, Eppendorf Himac Technologies Co., Ltd, Japan).

### Characterization of Aqueous Polymer Dispersions

Interfacial tension was determined through pendant drop shape analysis using a contact angle meter (DMs‐401, Kyowa Interface Science) at 40 °C and 80% relative humidity. The mass densities of the samples were assessed using a benchtop density meter (DMA4501, Anton Paar). The characterization was conducted at consistent temperatures.

### Drying Experiments for Statistical Analysis

The aqueous dispersion at ∼25 °C was transferred into a top‐side open cell, specifically the Hele–Shaw cell. These cells were then placed in an oven at a constant temperature under atmospheric pressure using the air circulator of a forced convection system (AS ONE, OFWP‐600 V). Given that the volume of the oven (600 mm × 497 mm × 500 mm, ∼150 L) with the air circulator exceeded that of the samples (< 1 mL), the relative humidity in the oven was regulated by the designated temperature.

### Drying Experiments for Spatiotemporal Analysis

The sample in the cell was placed in a ∼1 L observation box under controlled temperature and humidity using a temperature‐controlled air circulation pump. The internal temperature of the box was controlled using two methods: the first involved connecting a heat pen to a circulating tube, and the second involved attaching a rubber heater to the inner walls of the box. The relative humidity in the box was controlled by maintaining a constant temperature and ensuring air circulation. The correlation between the temperature and relative humidity was verified, confirming the ability to sustain consistent humidity during the drying experiments. Throughout the drying process, the samples were observed through glass windows, and images were captured at certain time intervals (10 min). The interfacial positions (X, Z) were determined by establishing the top corner of the cell as the coordinate origin (O).

## Conflict of Interest

The authors declare no conflict of interest.

## Author Contributions

T.K.L.N. and K.Ok. wrote the manuscript. T.K.L.N., T.H., K.Og., and Y.T., carried out the experiments. All authors contributed to the interpretation. K.Ok. supervised the project. All authors reviewed the manuscript.

## Supporting information



Supporting Information

Supplemental Movie 1

Supplemental Movie 2

## Data Availability

The data that support the findings of this study are available in the supplementary material of this article.

## References

[1] Q. Zhang , M. A. Amooie , M. Z. Bazant , I. Bischofberger , Soft Matter 2021, 17, 1202. 33427833 10.1039/d0sm01706j

[2] Q. Zhang , S. Zhou , R. Zhang , I. Bischofberger , Sci. Adv. 2023, 9, abq6820. 10.1126/sciadv.abq6820 PMC9839321 36638169

[3] A. R. Palmer , Science 2004, 306, 828. 15514148 10.1126/science.1103707

[4] B. Li , F. Jia , Y. P. Cao , X. Q. Feng , H. Gao , Phys. Rev. Lett. 2011, 106, 234301. 21770507 10.1103/PhysRevLett.106.234301

[5] R. D. Deegan , O. Bakajin , T. F. Dupont , G. Huber , S. R. Nagel , T. A. Witten , Phys. Rev. E 2000, 62, 756. 10.1103/physreve.62.756 11088531

[6] Y. Sang , M. Liu , Symmetry 2019, 11, 950.

[7] C. J. Gibb , J. Hobbs , D. I. Nikolova , T. Raistrick , S. R. Berrow , A. Mertelj , N. Osterman , N. Sebastián , H. F. Gleeson , R. J. Mandle , Nat. Commun. 2024, 15, 5845. 38992039 10.1038/s41467-024-50230-2 PMC11239904

[8] A. Herczyński , R. Zenit , Symmetry 2024, 16, 621.

[9] N. Stoop , R. Lagrange , D. Terwagne , P. M. Reis , J. Dunkel , Nat. Mater. 2015, 14, 337. 25643032 10.1038/nmat4202

[10] M. Tagliazucchi , E. A. Weiss , I. Szleifer , Proc. Natl. Acad. Sci. USA 2014, 111, 9751. 24958868 10.1073/pnas.1406122111 PMC4103377

[11] M. Fialkowski , K. J. M. Bishop , R. Klajn , S. K. Smoukov , C. J. Campbell , B. A. Grzybowski , J. Phys. Chem. B 2006, 110, 2482. 16471845 10.1021/jp054153q

[12] P. Kumar , D. Horváth , Á. Tóth , Soft Matter 2023, 19, 4137. 37249219 10.1039/d3sm00461a

[13] I. Bihi , M. Baudoin , J. E. Butler , C. Faille , F. Zoueshtiagh , Phys. Rev. Lett. 2016, 117, 034501. 27472115 10.1103/PhysRevLett.117.034501

[14] W. Song , N. N. Ramesh , A. R. Kovscek , Colloids Surf. A 2020, 584, 123943.

[15] A‐D. C. Nguindjel , P. J. de Visser , M. Winkens , P. A. Korevaar , Phys. Chem. Chem. Phys. 2022, 24, 23980. 36172850 10.1039/d2cp02542f PMC9554936

[16] A. Eslami , R. Basak , M. S. Taghavi , Ind. Eng. Chem. Res. 2020, 59, 4119.

[17] R. X. Suzuki , H. Tada , S. Hirano , T. Ban , M. Mishra , R. Takedaa , Y. Nagatsu , Phys. Chem. Chem. Phys. 2021, 23, 10926. 33912869 10.1039/d0cp05810f

[18] T. Islam , P. S. Gandhi , Sci. Rep. 2017, 7, 16602. 29192191 10.1038/s41598-017-16830-3 PMC5709420

[19] R. X. Suzuki , Y. Nagatsu , M. Mishra , T. Ban , Phys. Rev. Fluids 2019, 4, 104005.

[20] F. Gallaire , P.‐T. Brun , Phil. Trans. R. Soc. A 2017, 375, 20160155. 28373378 10.1098/rsta.2016.0155 PMC5379038

[21] J. F. Rudge , D. McKenzie , J. Fluid Mech. 2024, 989, A2.

[22] E. Dickinson , Adv. Colloid Interface Sci. 2013, 199–200, 114. 10.1016/j.cis.2013.07.002 23916723

[23] A. A. Eljaouhari , R. Müller , M. Kellermeier , K. Heckmann , W. Kunz , Langmuir 2006, 22, 11353. 17154625 10.1021/la061152w

[24] X. Liu , G. Farahi , G. L. Chiu , Z. Papic , K. Watanabe , T. Taniguchi , M. P. Zaletel , A. Yazdani , Science 2022, 375, 321. 34855512 10.1126/science.abm3770

[25] K. Okeyoshi , M. K. Okajima , T. Kaneko , Biomacromolecules 2016, 17, 2096. 27077450 10.1021/acs.biomac.6b00302

[26] K. Okeyoshi , Polym. J. 2020, 52, 1185.

[27] P. G. de Gennes , F. Brochard‐Wyart , D. Quéré , Capillarity and Wetting Phenomena: Drops, Bubbles, Pearls, Waves; Springer New York, NY, NY, USA, 2003.

[28] D. G. A. L. Aarts , J. Phys. Chem. B 2005, 109, 7407. 16851848 10.1021/jp044312q

[29] K. Okeyoshi , M. Yamashita , K. Budpud , G. Joshi , T. Kaneko , Sci. Rep. 2021, 11, 767. 33436957 10.1038/s41598-020-80779-z PMC7804455

[30] J. Yong , F. Chen , Q. Yang , J. Huo , X. Hou , Chem. Soc. Rev. 2017, 46, 4168. 28462414 10.1039/c6cs00751a

[31] D. V. Antonov , A. G. Islamova , P. A Strizhak , Materials 2023, 16, 5932. 37687631 10.3390/ma16175932 PMC10488358

[32] W. Feng , E. Ueda , P. A. Levkin , Adv. Mater. 2018, 30, 1706111. 10.1002/adma.201706111 29572971

[33] B. Mondal , M. M. G. Eain , Q. F. Xu , V. M. Egan , J. Punch , A. M Lyons , ACS Appl. Mater. Interfaces 2015, 7, 23575. 26372672 10.1021/acsami.5b06759

[34] L. Fei , Z. He , D. J. LaCoste , H. T. Nguyen , Y. Sun , A. Mini , Chem. Rec. 2020, 20, 1257. 32959509 10.1002/tcr.202000075

[35] L. Bai , L. Liu , M. Esquivel , B. L. Tardy , S. Huan , X. Niu , S. Liu , G. Yang , Y. Fan , O. J. Rojas , Chem. Rev. 2022, 122, 11604. 35653785 10.1021/acs.chemrev.2c00125 PMC9284562

[36] H. Zargartalebi , S. H. Hejazi , A. Sanati‐Nezhad , Nat. Commun. 2022, 13, 3085. 35654770 10.1038/s41467-022-30660-6 PMC9163176

[37] M. Li , L. Jiang , X. Li , T. Li , P. Yi , X. Li , L. Zhang , L. Li , Z. Wang , X. Zhang , A. Wang , J. Li , ACS Appl. Mater. Interfaces 2024, 16, 23904. 38684027 10.1021/acsami.4c02749

[38] M. Gericke , A. J. R. Amaral , T. Budtova , P. D. Wever , T. Groth , T. Heinze , H. Höfte , A. Huber , O. Ikkala , J. Kapuśniak , R. Kargl , J. F. Mano , M. Másson , P. Matricardi , B. Medronho , M. Norgren , T. Nypelö , L. Nyström , A. Roig , M. Sauer , H. A. Scholst , J. v. d. Linden , T. M. Wrodnigg , C. Xu , G. E. Yakubov , K. S. Kleinschek , P. Fardim , Carbohydr. Polym. 2024, 326, 121633. 38142079 10.1016/j.carbpol.2023.121633

[39] I. Mirza , S. Saha , ACS Appl. Bio Mater. 2020, 3, 8241. 10.1021/acsabm.0c01075 35019601

[40] A. Etale , A. J. Onyianta , S. R. Turner , S. J. Eichhorn , Chem. Rev. 2023, 123, 2016. 36622272 10.1021/acs.chemrev.2c00477 PMC9999429

[41] K. Budpud , K. Okeyoshi , M. K. Okajima , T. Kaneko , Small 2020, 16, 2001993. 10.1002/smll.202001993 32519469

[42] I. Saito , L. Wu , M. Hara , Y. Ikemoto , T. Kaneko , K. Okeyoshi , ACS Appl. Polym. Mater. 2022, 4, 7054.

[43] T. K. L. Nguyen , Y. Tonomura , N. Ito , A. Yamaji , G. Matsuba , M. Hara , Y. Ikemoto , K. Okeyoshi , Langmuir 2024, 40, 11927. 38821491 10.1021/acs.langmuir.4c00273 PMC11171445

[44] T. D. Nguyen , B. U. Peres , R. M. Carvalho , M. J. MacLachlan , Adv. Funct. Mater. 2016, 26, 2875.

[45] K. Okeyoshi , M. K. Okajima , T. Kaneko , Sci. Rep. 2017, 7, 5615. 28733650 10.1038/s41598-017-05883-z PMC5522447

[46] M. Chen , J. Chen , S. Liu , D. He , Y. Wang , H. Chen , D. Jin , X. Ma , Adv. Funct. Mater. 2024, 34, 2411036.

[47] R. Wang , Y. Feng , D. Li , K. Li , Y. Yan , Green Chem. 2024, 26, 9075.

[48] K. Okeyoshi , G. Joshi , M. K. Okajima , T. Kaneko , Adv. Mater. Inter. 2018, 5, 1701219.

[49] G. Joshi , K. Okeyoshi , T. Mitsumata , T. Kaneko , J. Colloid Interf. Sci. 2019, 546, 184. 10.1016/j.jcis.2019.03.062 30913492

[50] L. Wu , I. Saito , K. Hongo , K. Okeyoshi , Adv. Mater. Inter. 2023, 10, 2300510.

[51] G. Rajchakit , R. Sriraman , N. Boonsatit , P. Hammachukiattikul , C. P. Lim , P. Agarwal , Adv. Differ. Equ. 2021, 208, 2021.

[52] G. Rajchakit , R. Sriraman , N. Boonsatit , P. Hammachukiattikul , C. P. Lim , P. Agarwal , Adv. Differ. Equ. 2021, 256, 2021.

[53] X. Wang , J. Shi , G. Zhang , J. Math. Anal. Appl. 2021, 497, 124860. 10.1016/j.jmaa.2020.124850 PMC7733552 33343038

[54] E. N. M. Cirillo , R. Lyons , A. Muntean , S. A. Muntean , arXiv 2024,2405.16459v1.

