# Supplementary material for: Symmetry Breaking in Meniscus Splitting: Effects of Boundary Conditions and Polymeric Membrane Growth
Source: Adv Sci (Weinh). 2025 Jun 3;12(32):e03807. doi: 10.1002/advs.202503807 (PMC12407357; doi:10.1002/advs.202503807)
Supplement: Supplementary file 1 — Supporting Information [file ADVS-12-e03807-s001.docx]

Supporting Information

Symmetry breaking in meniscus splitting: Effects of boundary conditions and polymeric membrane growth

Thi Kim Loc Nguyen, Taisuke Hatta, Koji Ogura, Yoshiya Tonomura, Kosuke Okeyoshi^*^


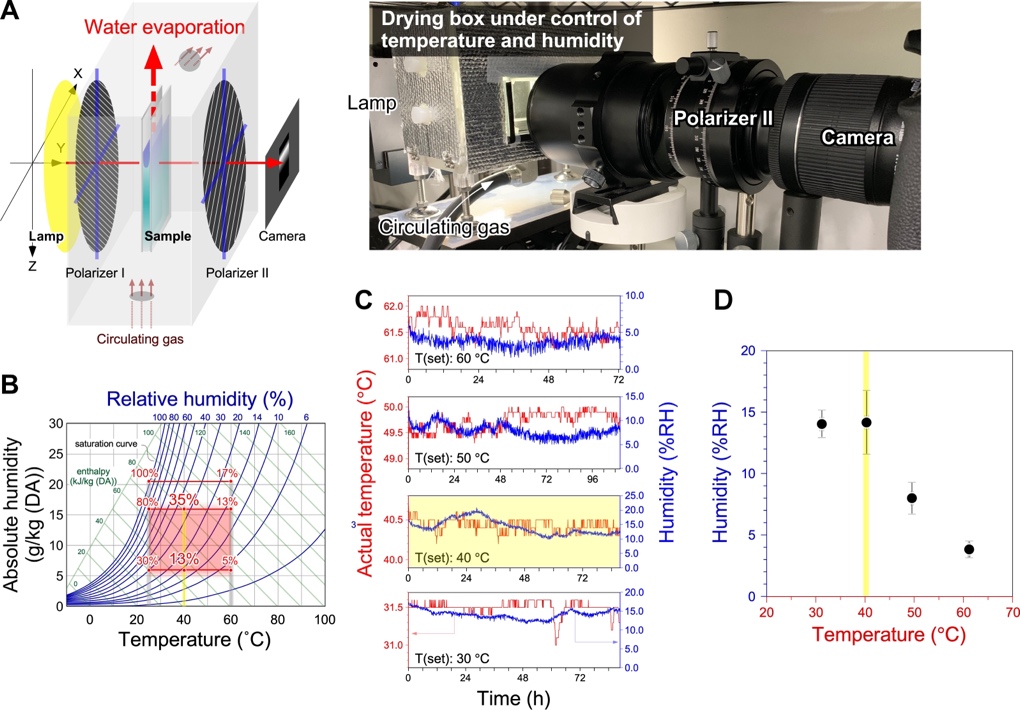


**Figure S1. A.** Experimental setup for drying tests and observations under cross-polarized light. The polarizers were set to 45° and 135°. **B.** Standard Mollier diagram for the estimation of the relationship between the temperature and humidity. DA = dry air. **C.** Time course of the actual temperature and relative humidity (RH) in a drying box at a given set temperature. **D.** Relationship between the temperature and RH in a drying box. By measuring the time course of the actual temperature and humidity in the drying box, the RH at 40 °C was estimated to be 14.2 ± 2.5% RH.

**Figure S1** presents a comprehensive analysis of the drying conditions to ensure accuracy and reproducibility. These measurements, based on the actual data from the drying box, provide a robust foundation for the experimental conditions and reinforce the reliability of the reported results.


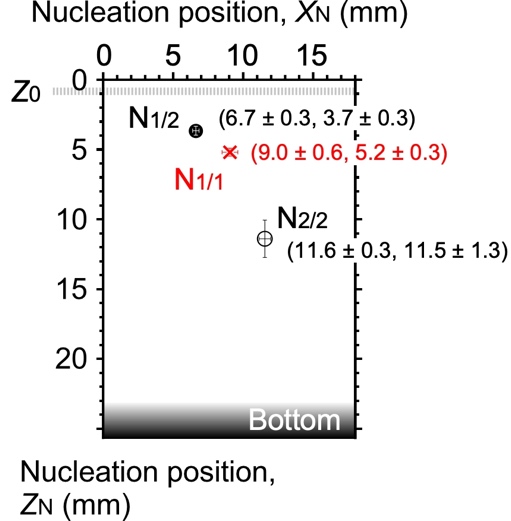


**Figure S2.** Statistical average of the nucleation position (*X*_N_, *Z*_N_) for cases with one nucleus and two nuclei in a cell (dimensions of 18 mm × 0.5 mm × ~23 mm). N_1/1_ represents the nucleus from a single nucleation, while N_1/2_ and N_2/2_ represent the first and second nuclei, respectively, for the case of two nucleations.

The preparation of the chitosan dispersion by the meniscus splitting method was conducted under the following conditions: initial chitosan concentration of 2.5 wt%, initial acetic acid concentration of 1.0 vol%. The cell had a width of 18 mm, a gap of 0.5 mm, and a depth of ~23 mm. The drying temperature was set to 40 °C, and the relative humidity was estimated to be 14.2 ± 2.5% RH (**Movie S1** and **Movie S2**, MOV). After pouring the chitosan solution into the cell, the cell was placed in the drying oven. The liquid in the samples was mostly evaporated in approximately three days.


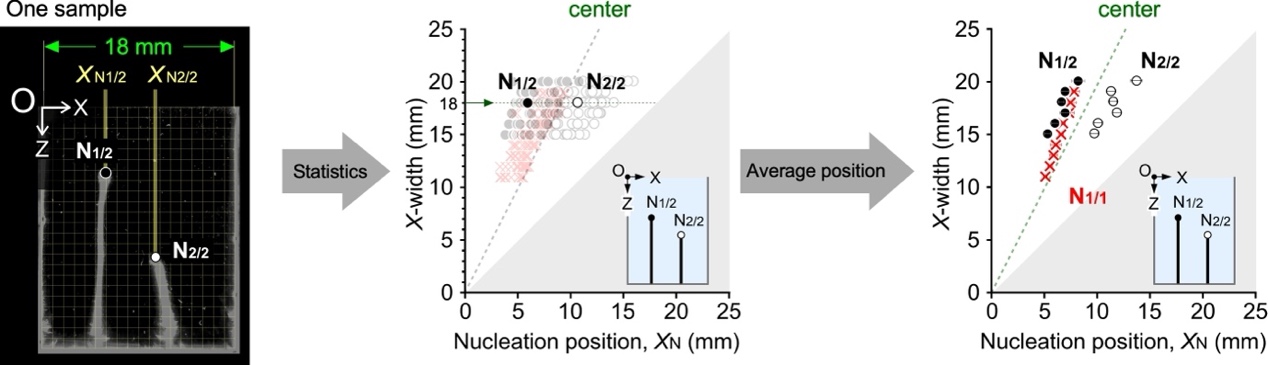


**Figure S3.** Procedure for the statistical analysis.


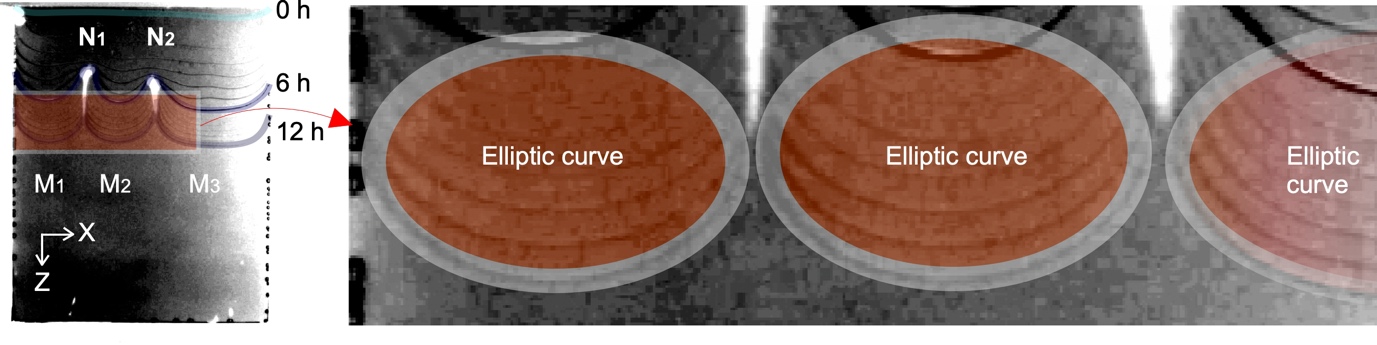


**Figure S4.** Time-lapse observation of meniscus splitting. Initial chitosan concentration: 2.5 wt%. Initial acetic acid concentration: 1.0 vol%. Cell dimensions: 18 mm, 0.5 mm, ~23 mm. Drying temperature: 40 °C.
